# Efficient generation of receiver operating characteristics for the evaluation of damage detection in practical structural health monitoring applications

**DOI:** 10.1098/rspa.2016.0736

**Published:** 2017-03-22

**Authors:** Chang Liu, Jacob Dobson, Peter Cawley

**Affiliations:** Imperial College, London, UK

**Keywords:** structural health monitoring, guided wave ultrasonics, receiver operating characteristic, damage detection, component analysis, pipe monitoring

## Abstract

Permanently installed guided wave monitoring systems are attractive for monitoring large structures. By frequently interrogating the test structure over a long period of time, such systems have the potential to detect defects much earlier than with conventional one-off inspection, and reduce the time and labour cost involved. However, for the systems to be accepted under real operational conditions, their damage detection performance needs to be evaluated in these practical settings. The receiver operating characteristic (ROC) is an established performance metric for one-off inspections, but the generation of the ROC requires many test structures with realistic damage growth at different locations and different environmental conditions, and this is often impractical. In this paper, we propose an evaluation framework using experimental data collected over multiple environmental cycles on an undamaged structure with synthetic damage signatures added by superposition. Recent advances in computation power enable examples covering a wide range of practical scenarios to be generated, and for multiple cases of each scenario to be tested so that the statistics of the performance can be evaluated. The proposed methodology has been demonstrated using data collected from a laboratory pipe specimen over many temperature cycles, superposed with damage signatures predicted for a flat-bottom hole growing at different rates at various locations. Three damage detection schemes, conventional baseline subtraction, singular value decomposition (SVD) and independent component analysis (ICA), have been evaluated. It has been shown that in all cases, the component methods perform significantly better than the residual method, with ICA generally the better of the two. The results have been validated using experimental data monitoring a pipe in which a flat-bottom hole was drilled and enlarged over successive temperature cycles. The methodology can be used to evaluate the performance of an installed monitoring system and to show whether it is capable of detecting particular damage growth at any given location. It will enable monitoring results to be evaluated rigorously and will be valuable in the development of safety cases.

## 1. Introduction

Guided wave testing has been widely used to detect damage in structures, mainly due to its ability to cover relatively large areas of the structure and provide good defect localization capability with a reduced number of sensors [1]. Recent advances in sensing and computation technology [2] make it attractive to use permanently installed transducers to monitor the integrity of structures, which can potentially improve the reliability and reduce the operating cost associated with regular inspections [3–5]. In structural health monitoring (SHM), we often seek to detect the progression of damage that develops over an extended period of time, such as a corrosion patch or a fatigue crack. In principle, the SHM approach enables smaller defects to be found than is possible in a one-off inspection, and for defects to be found more reliably in the vicinity of structural features. However, although extensive research has been done on inspection reliability, there is not yet a robust and efficient methodology for the evaluation of the performance of an SHM system in its real, practical implementation.

In long-term monitoring, damage is usually detected by comparing measurements with baseline records and seeking changes that represent defect signatures. The comparison can be done by subtracting the baseline from the current measurement [3], by calculating the cross-correlation between the current measurements and the baseline [6], or by more advanced data-driven methods such as wavelet transform [7] or component analysis [8,9]. If damage were the sole source of change in the duration of the monitoring, such a comparison would accurately reveal the progression of damage. However, guided wave records in long-term monitoring are often affected by various environmental and operational conditions (EOCs), which degrade the performance of the damage detection [10,11]. Therefore, it is critical to predict the performance of damage detection schemes under practical EOCs if the method is to find widespread use, and this paper presents a methodology for achieving this.

Conventional evaluation methods, such as a probability of detection (POD) analysis [12–14], require data from laboratory experiments under varying EOCs and physically growing defects on realistic structures. To evaluate an inspection scheme, we can first create damage on structures and then measure the structures under varying EOCs. However, in SHM, the performance may change with different histories of the EOC variations and also over the damage progression. Therefore, the two effects are coupled and need to be sampled simultaneously in order for us to assess the performance. To achieve this, we need to create damage on many structures, which is often not practical, or at least very inefficient, because we are usually interested in monitoring large areas. Hence, we need an alternative way to assess the performance of detecting damage progression using an SHM system in its practical setting. The new approach has to be robust to practical EOC variations, and capable of dealing with different damage evolution cases.

Modern computational resources mean that whereas when long range guided wave inspection was in its infancy it was only possible to do two-dimensional simulations [15], in the last few years full three-dimensional simulations of realistic corrosion patches has become feasible [16]. The three-dimensional simulations are now very efficient with the advent of graphics card processing schemes [2]. However, reliable prediction of signal changes due to environmental and other variability is much more difficult, because of the complexity of these effects [10,11,17]. On the other hand, obtaining experimental data with environmental variation from an undamaged structure is easy. Therefore, we propose a methodology of measuring data over multiple environmental cycles on an undamaged structure and synthetically adding damage to the signals.

This hybrid approach enables us to add damage at different locations with different growth patterns, and to easily investigate the effects of other practical parameters such as the degree of EOCs, damage severity and location, frequency of readings, etc. Also, by repetitively randomly selecting from the records that are collected in specific EOC ranges, we can gather sufficient data to statistically evaluate the performance of different damage detection schemes in these practical settings. The statistical measure we use to assess the damage detection is the receiver operating characteristic (ROC) curve, which is used in statistics to illustrate the performance of a binary classifier [18], and has also been widely adopted in non-destructive evaluation/SHM [19,20]. We will use the proposed framework to assess the performance of damage growth tracking using the baseline subtraction method, as well as the more advanced component analysis methods, singular value decomposition (SVD) and independent component analysis (ICA).

The methodology can be applied to any guided wave inspection system but in order to make the processing involved clearer, it is presented in the context of a controlled trial of a permanently installed monitoring system on a length of 8 inch diameter pipe that is subject to temperature cycling, as described in §2. In §3, we present the new ROC generation scheme and numerically validate the superposition involved using finite-element analysis. Section 4 reviews three damage detection schemes for SHM, namely baseline subtraction residual, SVD and ICA. These are compared using the proposed evaluation framework in §5 and an experimental validation is presented in §6.

## 2. Tests on a pipe monitoring system

The biggest commercial application of guided ultrasonic wave testing is for the periodic inspection of pipelines in the petrochemical industry and the power sector [1], where pipes are often buried, submerged or too high for access without scaffolding, making it difficult and expensive to gain access to even a single location along them. The main advantage of the method is its ability to periodically screen many metres of pipe from a remote location, resulting in substantial time and cost savings. Standard guided ultrasonic wave inspection works by transmitting a single guided wave mode (usually the torsional mode) and identifying the reflections from any discontinuities in the pipe. This approach assumes that each echo in the recorded signal is distinct from all others and is free from interference and coherent noise; in the case of pipelines, it is typically very effective due to the one-dimensional nature of these structures and their relatively low feature density. However, the ability to detect damage occurring at a feature such as a weld is limited because an echo is obtained even from a good weld, so damage is only seen if the signal can be identified as abnormal. In complex lines with multiple features such as tees, diameter changes, bends etc., the echoes from different features may overlap and interfere with each other, making reliable detection more difficult. Furthermore, the sensitivity of ultrasonic guided wave inspection to small discontinuities is limited, as it is necessary for a discontinuity to reflect a wave packet of greater magnitude than the underlying noise floor for it to be detectable. In this section, we demonstrate our experimental set-up to collect commercial quality guided wave signals from a length of plain pipe experiencing realistic variations in environmental condition.

**Figure 1.**
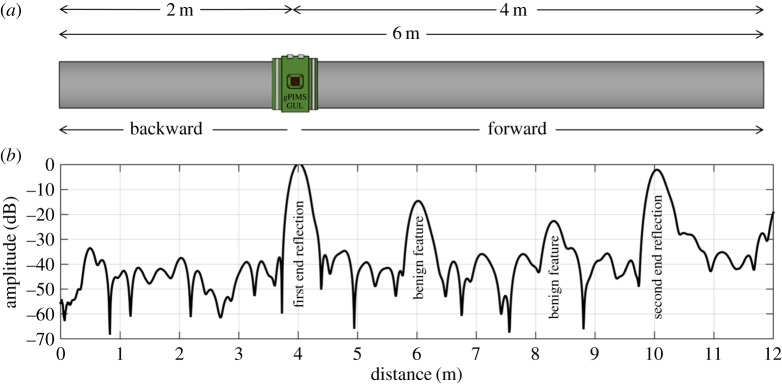
(*a*) Experimental set-up on a 6 m 8 inch schedule 40 steel pipe instrumented with a Guided Ultrasonics, Ltd. permanently installed monitoring system (PIMS). (*b*) A typical T(0, 1) mode, forward-direction A-scan of the pipe including reverberations. (Online version in colour.)

Figure 1*a* shows a schematic drawing of the experimental set-up on a 6 m long, 8 inch schedule 40 carbon steel pipe (wall thickness 8.2 mm). A resistive heating element was inserted and suspended in the centre of the pipe to uniformly cycle the temperature of the pipe. A commercial guided wave sensor (gPIMs unit manufactured by Guided Ultrasonics Ltd) was installed on the outside of the pipe, 2 m from one end; this sends and receives the torsional (T(0, 1)) mode in the pipe. The whole set-up was then wrapped in an insulating material. We use the experimental setup to investigate the effect of temperature variations, which is one of the main contributors to signal changes in guided wave monitoring, on commercial quality guided wave monitoring signals.

In each temperature cycle, the pipe was heated to 90°*C*, held to allow the temperature to equalize throughout the pipe and then allowed to naturally cool to 30°*C* in about 10 h. Guided wave measurements were taken every 15 min, resulting in about 40 measurements per cycle, covering a range of temperatures. Over 1100 experimental signals were measured over 26 temperature cycles. The excitation used in this analysis was an eight-cycle Hanning-windowed toneburst with a centre frequency of 23.5 kHz. The data used in this paper were the same as that in [21] that looked at a different signal processing method.

Figure 1*b* shows the envelope of a typical forward-direction (defined in figure 1*a*) T(0, 1) mode A-scan; distance is measured from the sensor, which is located at 0 m. Note that the distance axis is computed by multiplying the wave velocity by half the arrival time because the wave travels from the transmitter to the feature and back. The signal is plotted on a decibel scale, and is normalized such that the amplitude of the first cut end reflection, seen at 4 m, is 0 dB. Note that we consider even those parts of the signal which indicate a distance further than 4 m. These are not caused by physical features beyond the end of the pipe, but arise due to reverberations between the pipe ends and interactions with the transducer ring. These signal components serve as a useful model for a longer pipe with multiple features, such as welds, supports or bends.

## 3. Evaluation framework for structural health monitoring system

### (a) Generic scheme

**Figure 2.**
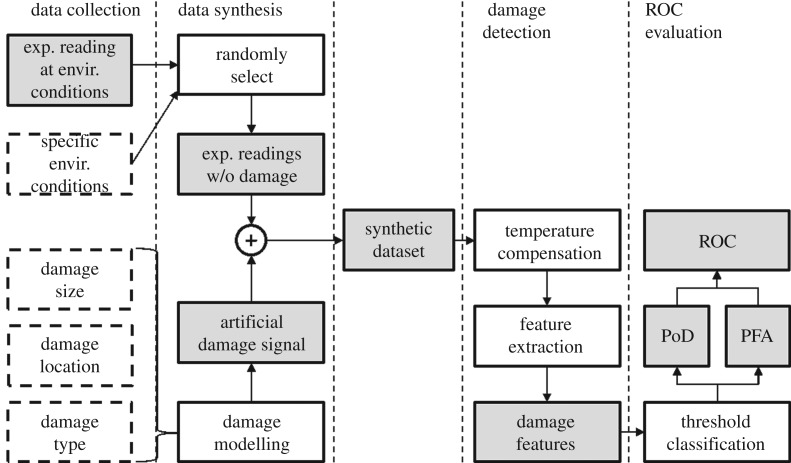
Schematic of the proposed evaluation framework with dashed boxes indicating parameters, dark-shaded boxes indicating raw/processed data and white boxes indicating data operations. Synthetic datasets are generated using undamaged experimental signals and predicted damage signals. The ROC curve is then generated after damage detection is performed on the synthetic datasets.

We propose an evaluation framework using experimental data over multiple environmental cycles on an undamaged structure and synthetic damage signatures. Figure 2 shows the flow diagram of the proposed methodology. We first collect experimental data on an undamaged structure while varying environmental and operational conditions (EOCs) that are likely to occur in its operation. Such data collection can be completed in a relatively short time by physically applying EOC variations that may occur more slowly in industrial operation, and setting the monitoring interval accordingly. The experimental data then serves as the undamaged monitoring signals that would be seen in the absence of damage growth.

We then specify the EOCs of interest and randomly select from experimental records collected under such conditions. For each test scenario, we repeat the randomization process multiple times to generate multiple synthetic datasets in order to sample from the possible histories of EOCs. We then superpose artificial damage signals to each of the undamaged datasets to create the synthetic datasets, with the damage following a specified growth pattern. These datasets then enable the mean and variability of the ROC to be determined.

The generated synthetic datasets are first compensated for any temperature variations, using a stretch-based temperature compensation algorithm [22], then processed to extract any damage features. Three damage feature extraction methods, namely, baseline subtraction residual, SVD and ICA, are implemented and compared in this study, as detailed in §4. We create synthetic datasets at different EOC and damage conditions, and compute the ROC curves for the different methods under different degrees of EOC variation and at different levels of damage growth.

Section 3b illustrates the process to predict damage signals and superpose them at the locations of interest. In order to show that the synthetic dataset can reliably represent the signals from guided wave interaction with real damage on a pipe, §3c compares the superposition of localized damage on noisy signals generated from generally corroded pipe with full finite-element analysis of the same localized damage plus the general corrosion.

### (b) Prediction of artificial damage signal and superposition onto undamaged signals

Unlike conventional one-off inspection, in SHM we process a large set of monitoring records collected over time, and seek to identify a progressive trend of damage growth, even if the absolute damage amplitude is small. In our evaluation framework, we create synthetic datasets to mimic this process. In this paper, we represent growing corrosion by the constant growth rate of the cross-sectional area loss (CSAL) due to flat bottom holes of increasing cross-sectional area loss with a constant diameter-to-depth ratio of two. We linearly increase CSAL from zero to 3%, in order to simulate damage growth, and compute the diameters and depths of the flat-bottom holes during the progression accordingly.

We predict the damage signature by first generating reflection coefficients of flat bottom holes of growing size on a homogeneous plate using the analytical model developed in [23], modified to compensate for the curvature of the pipe geometry [16]. Then at each time step during the damage progression, we multiply the reflection coefficients from the hole by the Hanning-windowed inspection toneburst in the frequency domain; this frequency domain signal is then transformed into the time-domain by applying an inverse Fourier transform to obtain the reflection signals from the hole at that particular time. Note that although the CSAL increases linearly with time, the amplitude of the damage reflection increases nonlinearly and is frequency dependent [23].

To simulate damage at a location of interest, we compute the arrival time of the damage reflection knowing the damage location and then superpose the damage reflection onto the undamaged signals collected in the experimental set-up in §2. The undamaged signals are randomly selected from experimental signals collected over a range of EOCs, to mimic the monitoring records affected by temperature variations that could occur in the real world. The arrival time of damage is computed as a proportion of that of a significant structural feature, e.g. the second end reflection; this ensures that the damage reflection is inserted at the same spatial location in each record obtained at different temperatures. The damage reflections are then delayed and superposed on the undamaged experimental signals to create a synthetic dataset.

Table 1 summarizes the test parameters used to generate the synthetic datasets. Each synthetic dataset is generated with one value for each parameter. For example, a combination of 10°*C* temperature variation, 1% damage amplitude, 50 readings and 10.1 m means we generate a synthetic dataset that consists of 50 signals, where the undamaged signals are randomly selected from experimental records collected over a 10°*C* range; the 50 damage signals linearly increasing from 0 to 1% CSAL are predicted and added to the undamaged experimental signals at 10.1 m, slightly after the second cut end reflection.

**Table 1.** Test parameters used to generate synthetic data.

test parameter	value	unit
temperature range over which undamaged signals are selected	2, 10, 30, 60	°C
damage (CSAL) at the end of ramp	0.3,0.5,0.7,1.0,3.0	%
number of readings over which damage grows	10,30,50,70,100	—
damage location (away from features)	2.3, 7.5, 11.3	m
damage location (near to a feature)	6.2, 10.1	m

For each combination of test parameters, we repeat this synthetic data generation process 500 times. The resulting 500 synthetic datasets mimic the same damage progression but under different temperature histories, which enables us to statistically assess the damage detection performance in different practical settings. In total, about four million synthetic records are generated for 136 test scenarios with combinations of the test parameters shown in table 1.

Note that the temperature difference causes a significant change in the measured signals and the similarity between two signals collected at different temperatures drops quickly as the temperature difference between the two measurements increases. In our case when comparing two measurements with a 10°*C* temperature difference the correlation coefficient is about 0.4. Randomly ordering the randomly chosen measurements further decreases the correlation, in the same way that changing the order of components in a vector can produce a vector that is orthogonal to the original. Hence, by using measurements collected at different temperatures and by randomly ordering them in a dataset, we obtain datasets that are sufficiently different.

To ensure the independence of these synthetic datasets, we calculated the distance correlation coefficients [24] and the two-dimensional mutual information [25] between each pair of synthetic datasets generated for one test scenario. The distance correlation coefficient is similar to the Pearson correlation coefficient but is zero if and only if statistical independence is satisfied. The mutual information of two random variables *X* and *Y* measures the information they share, and can be expressed as *I*(*X*;*Y*)=*H*(*X*,*Y*)−*H*(*X* | *Y*)−*H*(*Y*  | *X*), where *H*(*X* | *Y*) and *H*(*Y*  | *X*) are the conditional entropies, and *H*(*X*,*Y*) is the joint entropy of *X* and *Y*. Both the distance correlation coefficients and the ratio of mutual information over joint entropy (*I*(*X*;*Y*)/*H*(*X*,*Y*)) are scalars in (0, 1), where zero means they are independent, and unity means one completely determines the other.

Both measures show weak dependence between datasets when the number of measurements in a dataset is greater than 10. For example, for 500 datasets each containing 50 measurements randomly drawn from a 30°*C* range, the 95% upper bound of the two independence measures were, respectively, 0.30 and 0.12. For all datasets having at least 30 measurements, the two independence measures are all below 0.4 and 0.14, respectively. When the number of measurements decreases to 10, the dependence between different datasets becomes much stronger, and the independence assumption no longer holds. Therefore, in the later sections, we only analyse the results using synthetic datasets with more than 10 measurements, which allows us to evaluate not only the mean but also the variance of the damage detection performance.

### (c) Finite-element validation of the superposition model

The framework described above relies on the superposition of predicted damage reflections onto measured signals from the undamaged structure. To validate this approach, a comparison was made between the signals generated using superposition and signals generated from a full finite-element model. Such a comparison can be used to confirm that the superposition is a suitable alternative to full experimental or modelling work. A comparison was made for three representative cases that approximate a range of potential inspection scenarios. The first comparison is for a plain section of pipe with a growing flat bottom hole defect. The second comparison is also for a plain section of pipe with a growing defect but with additional temperature variations as the defect grows, followed by processing of the data using a temperature compensation procedure. The final comparison is for a defect growing in a weld cap, again with significant temperature variations and temperature compensation.

**Figure 3.**
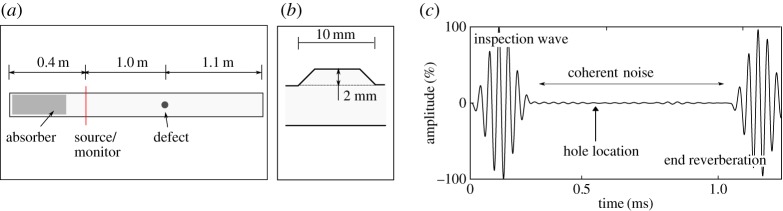
The model used in the validation showing (*a*) the model set-up, (*b*) detail of the weld structure and (*c*) a typical time trace from the model, with main signal features identified. (Online version in colour.)

The finite-element modelling was based on the pipe specimen described in §2 with the dimensions of a 6 inch schedule 40 pipe (outer diameter 168 mm and wall thickness 7 mm). For efficiency, we modelled a smaller-diameter pipe and did not model the whole length of the pipe; instead, this cylinder was 2.4 m long and was discretized with 2 mm characteristic length tetrahedral elements. The resulting elements were given the properties of mild steel, with these properties modelled as temperature dependent according to [26]. Superposition would not be required on a plain pipe giving no reflected signal in the absence of a defect because it would only be necessary to predict the defect reflection. However, in practice there is coherent noise on the signals and defects can occur at pre-existing reflectors such as welds. It is therefore necessary to check the validity of superposition in these cases. In order to create a model with coherent noise the inner surface of the pipe was perturbed to give a Gaussian profile, generated and applied to the model in the same way as in [27]. This rough surface had a mean depth of 2 mm and a correlation length of 50 mm and scattered the incoming wave, so generating the required coherent noise. The coherent noise produced had a maximum level typically around 2.0% of the inspection wave amplitude, which is consistent with coherent noise levels seen in commercial inspections. It should be stressed that there are many sources of coherent noise in practice; a rough surface was used here as a convenient means of generating it in simulations. The pipe was then set up with absorbing regions following Pettit *et al*. [28], a ring of source and monitoring nodes 0.4 m from the pipe end and a defect introduced in certain models. This defect was modelled as a flat bottom hole in the pipe outer wall, 1.0 m from the guided wave source. The cross-section of this hole varied from 0.2% to 2.0% of the pipe cross-section but had a constant diameter to depth ratio of 2:1. In some cases, a weld was also included and in such cases was modelled as a thickness increase with a ramp–flat–ramp profile, a total length of 10 mm and a height of 2 mm starting at a distance of 0.995 m from the source of guided waves. In models with both a weld and a hole this means the hole was removing material from the weld cap. An example model of a plain pipe with a flat bottom hole is shown in figure 3*a* and the weld geometry is shown in figure 3*b*. These models were then solved using POGO [2], a GPU based finite-element solver and where temperature compensation was employed the procedure was the same as used elsewhere in this paper.

**Figure 4.**
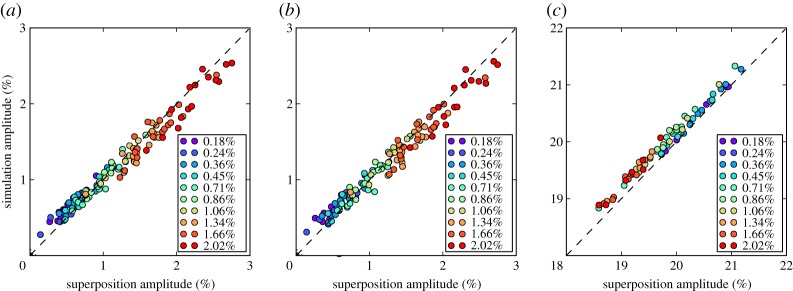
Comparison of signals from the full finite-element model with signals from superposition in different scenarios: (*a*) with general corrosion (*b*) with general corrosion and temperature variations and (*c*) with general corrosion, temperature variations and a weld. Key shows different CSAL—the % cross-section of pipe removed by hole. (Online version in colour.)

Data for the first comparison were generated from two models. The first model was for a pipe with a hole in the outer surface, initially with a cross-section of 0.2% while the second model was for a plain pipe. A rough surface was generated and the same surface applied to both models, which were then solved. Figure 3*c* shows an example time history for the model containing a hole which shows that the defect reflection is much smaller than surrounding coherent noise. Superposition was then used to add a defect signal to the time history for the undamaged pipe, with the superposed signal added at the same location as the modelled hole. A comparison was then made of the maximum amplitude in the time window 0.55–0.6 ms, in which the defect reflection appears. Maximum amplitude was used rather than other metrics because it is directly used in the damage detection algorithm. This process was then repeated for 10 different hole sizes, with 10 different rough surfaces used for each hole size to give a total of 100 data points. The results of this comparison are shown in figure 4*a*.

The modelling procedure for the second comparison was the same except the models were adjusted for temperature variations. For each pair of models a temperature was chosen at random from within the range 10–40°C and the material properties updated accordingly. Care was taken when performing superposition to insert the defect signal at the correct time; as temperature variations change the time of flight of ultrasonic waves the defect needed to be inserted at such a time as to ensure that the defect was in the same location. Before the comparison of amplitudes was done, the signals were stretched to 25°C using the temperature compensation procedure outlined in §4a; the results are given in figure 4*b*. The final comparison was exactly the same as the second except the model included a weld, which produced a reflection amplitude of around 20%, the results for this case are given in figure 4*c*.

The results show overall very good agreement between the superposition approach and full modelling. Perfect agreement would not be expected as the reflection from a hole of a given size is not strictly independent of the cross-section profile onto which it is placed; however, the trends of the full and superposition model results are similar so the superposition gives a satisfactory representation of the growth of the reflection with increasing cross-section loss. This agreement is largely independent of defect size and is not significantly affected by temperature changes and subsequent temperature compensation. The superposition approach also works as well at pipe features as it does in plain sections of pipe. Note that in figure 4*c* larger holes lead to a reduced signal amplitude because the hole is removing material from the weld (which is an increase in the cross-section). The net effect is to reduce the cross-sectional change in that region and hence the reflection.

### (d) Receiver operating characteristic

In statistics, ROC curves show the true positive rate (TPR=∑True Positive/∑Positive) against the false positive rate (FPR=∑False Positive/∑Positive), at various threshold values. In the field of NDE/SHM, ROC curves are also often used to illustrate the performance of damage detection, in which the two axes are termed the POD and the probability of false alarm (PFA), both in the range (0, 1).

A representative baseline subtracted residual signal obtained as discussed in §4a below is used here to illustrate the generation of ROC curves. We first randomly select 100 undamaged signals from the experimental data in §2, covering 10°C of temperature variation. Damage signatures of a linearly growing flat-bottom hole with maximum size of 1% are then predicted and added to the undamaged signals at 7.5 m, following the procedure of §3b. The temperature differences are first compensated using stretch-based scale-transform temperature compensation [22]; all 100 records are stretched or compressed to the baseline signal collected at the median temperature. The residual signals are then obtained using the procedure described in §4a.

**Figure 5.**
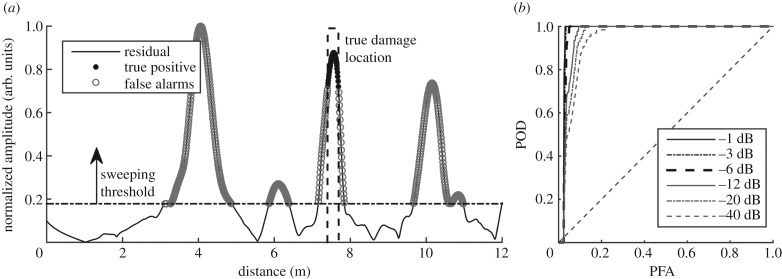
(*a*) Example residual signal obtained from a set of 100 measurements under temperature variation of 10°C, over which damage grows from zero to 1% of the cross-sectional area. (*b*) Receiver operating characteristic curves computed from the residual in (*a*) with different tolerance level.

Figure 5*a* plots the residual signal as a solid line, with its maximum amplitude normalized to unity; the dashed line shows the true damage location. As the excitation signal used in guided wave monitoring is typically a Hanning-windowed toneburst, we expect a reflection from features in the pipe to extend over at least this length in time; therefore, when we convert the time axis of the received signal to a length scale, the signals from features occur over a finite length of the pipe. Assuming a reflector has small axial extent compared with the wavelength, the reflected signal length will be approximately that of the input toneburst so we would expect to see a signal over this distance; the damage location on figure 5*a* is therefore shown as a finite length of pipe that is much larger than the true damage extent. To calculate the ROC, we sweep the threshold (dot-dashed line) from 0 to 1, and classify all the values above the threshold as positives. The positives that fall within the region expected for the true damage are labelled as true positives or detection (solid circles), and the positives elsewhere are labelled as false alarms (hollow circles). Note that our definition of positive is different from the classic binary (hit/miss) definition in that the location of the positives also matters. This is to be consistent with the real world practice where only detecting a feature at the damage location is considered a true detection; detecting features at wrong locations should be considered false alarms. At every threshold, we calculate the POD and false alarm rate, which becomes one point on the ROC curve. The collection of the points becomes the ROC curve for this test scenario.

In guided wave monitoring, damage can be masked by large residuals caused by EOC variations, even after compensation; this problem is most severe where there is a large signal in the undamaged structure, i.e. at structural features. In guided wave inspection practice, the inspector assesses the whole test length for defects and the POD and false call rates are defined over the whole length. Here, we are interested in monitoring the growth of damage as multiple readings will be obtained as it develops, but we retain the concept of assessing the whole pipe with one criterion. This is a harsh measure for feature-free areas because many more false calls are generated at the locations of features; it would be possible to segment the pipe and compare performance in similar regions. However, as the purpose of this paper is to set out the general methodology and to compare different feature extraction methods, this extra sophistication was not considered.

Since the excitation signal is a Hanning-windowed toneburst, the amplitude approaches zero at its beginning and end. Therefore, if we require detection over the whole theoretical length shown as the damage location in figure 5*a*, we will incur a high false call rate at the tails. We therefore say that we expect a defect to give a reflection above the ‘call level‘ over the spatial region where the signal is above a specified fraction (tolerance level) of the peak signal. We express this fraction in decibels so a −6 dB tolerance level corresponds to requiring the defect signal to be above the threshold at all points either side of the peak where the envelope of the input toneburst is above half its peak amplitude. If this criterion is met then the POD is unity; a lower tolerance level means we require more points above the threshold for unity POD. Thus, if the tolerance level is increased, the width of the dashed rectangle showing the ‘true’ damage location in figure 5 is reduced.

The ROC curve is an indicator of detection performance. The perfect detector yields a curve that goes through point (0, 1), indicating no false alarms and perfect detection (100% POD). A random guess yields a ROC curve following the 45° diagonal line, where the POD and PFA are equal. Figure 5*b* shows the ROC curves corresponding to different tolerance levels from −1 to −40 dB. As the tolerance level goes down, the less tolerant we are to noise, and the more false alarms we have at any given POD level. Here, we use a −6 dB tolerance level (corresponding to the ROC curve with a thicker dashed line) to be consistent with common industrial practice. It should be noted that the tolerance level defined here refers to the tolerance to noise seen in practical cases, which is different from the threshold that is swept from zero to unity to calculate POD and PFA.

## 4. Damage detection methodology for structural health monitoring

Using the proposed framework, we evaluate and compare three damage detection methods for guided-wave SHM. We first review the conventional baseline subtracted residual method in §4a, and two advanced data-driven methods, SVD and ICA, in §4b. Section 4c discusses the use of the Mann–Kendall test to identify a monotonic trend in the data.

These methods were chosen because they have been proposed previously and appeared promising. The framework proposed in this paper is designed to allow the evaluation of the performance of different damage detection methods in a SHM system in practical settings; we do not suggest that the methods applied here are the only possibilities. We simply seek to compare different possible processing methods, rather than attempting to define theoretical performance bounds. Finding a theoretical bound of performance may be possible for specific cases with many assumptions that may not always satisfactorily represent the real world environment; the biggest advantage of the proposed framework is that by using the undamaged signals collected from installed monitoring systems that are affected by the real-world EOCs, we can estimate the reliability of detecting a particular defect at a potential location.

### (a) Temperature compensation and baseline subtraction

One common method to enhance signal-to-noise ratio (SNR) for damage detection is baseline subtraction. By subtracting a baseline signal that is collected in a known undamaged state, the residual signal should in principle resemble the signature created by the defect, if one exists. Signals are often pre-processed before the subtraction to eliminate any difference between the current measurement and the baseline due to temperature change. Two groups of temperature compensation methods are commonly used in guided-wave testing, namely, optimal baseline selection [29] and optimal signal stretch [3,6,17]. In this paper, we implemented a stretch-based temperature compensation method [22], as these techniques allow comparison of all the measured signals with a single reference signal.

The stretch-based temperature compensation methods approximate the temperature change as a stretch or compression in time, and reverse the effect by first finding the optimal stretching factor that makes the measurement and the baseline most alike, and then stretching or compressing one to the other. Although the stretch approximation is usually valid when the temperature difference is small, the stretching can introduce frequency distortion when the temperature difference is large [30]. To minimize the distortion, we stretch measurements to a baseline collected at the median temperature to minimize the maximum temperature difference to be compensated. This can be achieved even without temperature measurements, because the estimated stretching factors are effectively a measure of the temperature.

In our processing of a synthetic dataset, we first stretch/compress all the measurements to the measurement at the median temperature in the particular dataset. Residual signals are then obtained by subtracting the first signal in the dataset from all the temperature compensated signals. As the reflection from small defects roughly replicates the inspection toneburst, we cross-correlate the obtained residual signals with the eight-cycle, Hanning-windowed toneburst to enhance the signal-to-noise ratio.

In conventional one-off inspection, the obtained residual signals are processed and assessed separately until a damage feature larger than a threshold is detected. However, in long-term monitoring we obtain many records over which the damage grows progressively, which can be used to improve sensitivity. Specifically, the residual at the damage location would change monotonically, while values elsewhere would not be due to random changes. If we can detect such a monotonic trend, we are more likely to identify the presence of damage at an earlier stage than in one-off inspection. In this paper, we consider the case where damage grows linearly from the start of monitoring. This is used to illustrate the method; it is not limited to this case.

The residual at location *x* can be written as a linear regression model of variable time *t*,

4.1 $$r_{xi}=\beta_{x0}+\beta_{x1}t_{i}+\epsilon_{i},$$

where *β*_*x*1_ is the rate of change at location *x* over time *t*, *β*_*x*0_ is the intercept corresponding to the residual at time zero, and *ϵ* is the error term which we assume follows the same distribution at all locations. The error consists of not only random system noise but also residual noise caused by imperfect compensation of the temperature differences, which follows a generalized Gaussian distribution if the monitoring period is sufficiently long and the temperatures at which readings are taken are randomly distributed.

We estimate the parameters *β*_*x*0_ and *β*_*x*1_ using least-squares linear regression. At locations where damage grows progressively, the slope *β*_*x*1_ represents the growth rate of damage over time; elsewhere, no systematic trend should exist so we expect *β*_*x*1_ to be close to zero. On the other hand, *β*_*x*0_ might not be zero because of imperfect baseline removal, but this is not connected to the progression of damage. Therefore, we can characterize the damage progression solely using the slope *β*_*x*1_, i.e. we use the values of *β*_*x*1_ at all locations along the pipe to represent the presence of damage and its progression if it is present.

The variance of the estimated damage growth rate *β*_*x*1_ is given by

4.2 $$\mathrm{Var}({\hat{\beta }}_{x1})=\frac{\sigma^{2}}{\sum_{i=1}^{N}{\left(t_{i}-\bar{t}\right)}^{2}},$$

where the numerator is the variance of the noise term *ϵ*, and the denominator is the variance of the time at which all the measurements are taken. Therefore, we are more confident about the estimated slope if more observations are taken, or measurements are taken over longer period of time, making this suitable for processing long-term monitoring data.

In conventional SHM practice, the residual is calculated by subtracting one-off measurements, which are subject to environmental variations. Here, we use least-square estimation (LSE) to estimate the growth rate so that the change caused by the environmental variations, being random in nature, can be cancelled out. This makes a fairer comparison of the residual method to the component analysis methods when many continuous measurements are available. Our results show that using the least-squares estimation gives better results than are obtained with conventional baseline subtraction.

### (b) Damage detection based on matrix decomposition

A recently developed group of methods is based on component analysis, which decomposes the data matrix into different components to represent variations produced by different sources. The damage reflections and the signals caused by other EOC variations vary with position and have different varying patterns over time, and will be separated into different components. By identifying the components related to damage, we effectively eliminate the variations from other sources and significantly improve the damage detection performance.

The general form of decomposition of long-term monitoring records is

4.3 $$\text{X}={\text{WA}}^{T},$$

where **X**, **W** and **A** are matrices that contain the measurements over time, the weights, and the components, respectively. **X** is the *N*×*D* data matrix that samples the structural status both spatially and temporally, where *N* is the number of signals measured over the monitoring period, and *D* is the number of sample points in each signal. The two dimensions correspond to two different sampling frequencies—the frequency at which measurements are acquired from the monitoring system (temporal sampling interval Δ*t*), and the sampling frequency of the A/D converter. The time axis of each measurement is converted to a distance scale by dividing the group velocity of the guided wave used by the sampling frequency to give a spatial sampling interval Δ*d*. Then, each column of **X** represents one record collected at time *t* from [0,Δ*t*,…,(*N*−1)Δ*t*], and each row represents the wave reflected from a certain location *d* from [0,Δ*d*,…,(*D*−1)Δ*d*]. The decomposition of **X** yields two matrices **W**^*N*×*R*^ and **A**^*D*×*R*^, each decomposing the variations along one dimension into *R* components, with *R*≪*N*,*D* representing the reduced dimensionality. Weight matrix **W** separates the variations over time, where each column **w**^*N*×1^ represents the trend over time corresponding to one source. Component matrix **A** separates the variations in space, where each column **a**^*R*×1^ represents the signal components along the distance axis.

The decomposition can be achieved in different ways. We implemented and compared two related decomposition methods, SVD and ICA [31]. The two methods are widely used in fields such as face recognition [32] and speech separation [33], and their application in SHM has been illustrated in [8] and [9]. The major difference between the two methods is the criteria by which the components are separated: SVD enforces the orthonormal of both the component and the weight matrices, while ICA maximizes the statistical independence only between the components.

**Figure 6.**
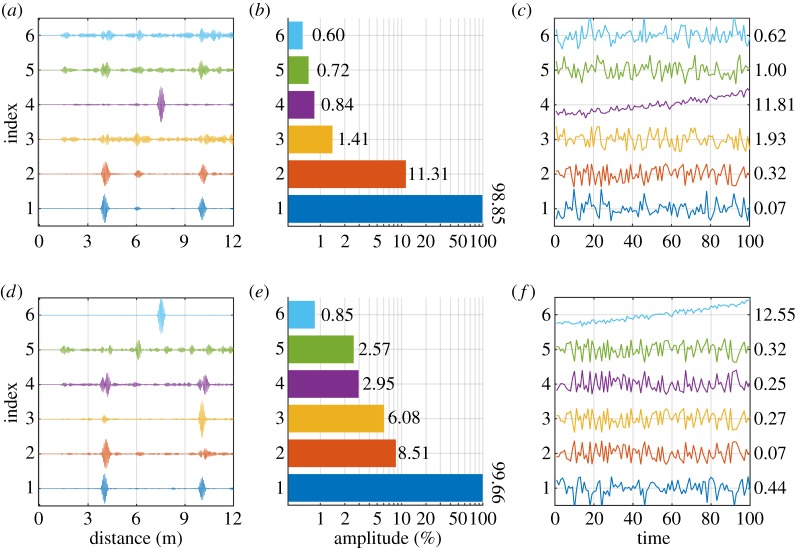
Decomposition of 100 synthetic records collected in a 10°C temperature range, over which damage grows linearly from 0 to 1% CSAL at 7.5 m. Six components from (*a*) SVD and (*d*) ICA are ordered by their absolute amplitudes shown in (*b*) and (*e*). The corresponding normalized weights over time (*c*) and (*f*) show the progressive trend over time. The MK test scores *Z*_*MK*_ (detailed in §4c) are labelled to the right of each set of weights. (Online version in colour.)

Figure 6 shows example results of applying SVD and ICA on the same synthetic dataset processed with the baseline subtraction residual method in §3d. The dataset contains 100 measurements with undamaged experimental signals over a temperature range of 10°C, superposed with artificial damage progressing to 1% CSAL at its maximum extent, and was pre-processed with the same temperature compensation procedure as in §3d and §4a. Both SVD and ICA decomposed the records into multiple components. We show in figure 6 the first six components and corresponding weights, as well as their absolute amplitudes at the end of the monitoring period.

Figure 6*a*–*c* shows the decomposition results using SVD, where figure 6*a* shows the first six normalized components plotted against distance from the sensor, ordered by their absolute amplitudes, which is shown in figure 6*b* on a logarithmic scale, and figure 6*c* shows their zero-centred and normalized weights over the 100 measurements. Figure 6*d*–*f* shows the corresponding results obtained using ICA.

The first components shown in figure 6*a*,*d* both resemble the undamaged A-scan of the pipe, with absolute amplitudes close to 100%. The corresponding weights in figure 6*c*,*f* vary randomly around the average amplitude. It should be stressed that the amplitudes of the weights shown in figure 6*c*,*f* are normalized to their individual maxima so that their trends can be seen; their relative magnitudes can be seen in figure 6*b*,*e*. The fourth SVD component in figure 6*a* and sixth ICA component in figure 6*d* are clearly related to damage, each showing a dominant peak at around 7.5 m where damage is added. The corresponding final amplitude of the two components, as shown in figure 6*b*,*e*, are 0.85% and 0.84%, which are consistent with the predicted reflection coefficient from the 1% CSAL defect at the end of the damage growth. Moreover, their corresponding weights over the 100 measurements in figure 6*c*,*f* clearly follow a monotonically increasing trend, with an increasing growth rate as CSAL progresses. This is consistent with our analytical model of a growing flat-bottom hole which predicts a nonlinear relation between the reflection coefficient and the CSAL [23,16].

Note that even with an increasing damage amplitude, the decomposed weights can decrease with time, in which case the phase of the corresponding component will shift by 180°. This is because ${\text{w}}_{i}{\text{a}}_{i}^{T}$ is equivalent to $\left(-{\text{w}}_{i})(-{\text{a}}_{i}^{T}\right)$, and both are valid results of the decomposition. Therefore, we can only identify the damage-related component by its monotonicity, not by its directionality. In §4c, we demonstrate the Mann–Kendall test, which we used to determine whether a monotonic trend (either increasing or decreasing) exists in the data.

The other components in figure 6*a*,*d* capture features at different locations, and have various amplitudes. They represent other sources of variation that exist in the data that are either orthogonal to (in the case of SVD) or statistically independent of (in the cases of ICA) the undamaged experimental signal and the damage signature. Note that the amplitudes of some other components are larger than that of the damage-related components. Nevertheless, the monotonic damage signatures are clearly distinguishable from the other components whose variation is either due to noise or related to the random temperature variation.

### (c) Identifying monotonic trend using Mann–Kendall trend test

The Mann–Kendall (MK) trend test [34,35] is a hypothesis test to determine whether a monotonic trend exists in the data. A monotonic trend means that the variable consistently increases or decreases through time, but the trend may or may not be linear. The MK test is a simple, non-parametric test that only requires the data to be distributed similarly over time, and does not depend on the magnitude of data or its exact distribution. The test and its variant forms are extensively used in the fields of geological and environmental studies [36]. Some variants of the test also take seasonal effects into consideration [37], which could potentially benefit SHM applications because the EOC affecting applications are often seasonal or cyclic.

The MK test assesses whether to reject the null hypothesis (*H*_0_: no monotonic trend) and accept the alternative hypothesis (*H*_*a*_: monotonic trend is present), where

4.4 $$\begin{array}{rl} & H_{0}:\mathrm{Prob}[x_{j}>x_{k}]=0.5\\ \text{and}\;\; & H_{a}:\mathrm{Prob}[x_{j}>x_{k}]\neq 0.5 \end{array}\;\forall j>k.\}$$

To evaluate the test, we first determine sign(*x*_*j*_−*x*_*k*_) for all *n*(*n*−1)/2 pairs of *j*, *k* where *j*>*k*, and compute *S* by,

4.5 $$S=\sum_{k=1}^{n-1}\sum_{j=k+1}^{n}\mathrm{sign}(x_{j}-x_{k}).$$

*S* essentially calculates the difference between the number of increases and the number of decreases. A positive *S* means the observations obtained later in time tend to be larger than observations made earlier. To determine whether the difference is statistically significant, we compute the MK test statistic *Z*_MK_,

4.6 $$Z_{\mathrm{MK}}=\frac{S-\mathrm{sign}(S)}{\sqrt{\mathrm{Var}(S)}},$$

where the variation of *S* [38] is computed as

4.7 $$\mathrm{Var}(S)=\frac{n(n-1)(2n+5)}{18}.$$

The *p*-value of the test statistic is then compared with the significance level *α*, which is the probability of wrongly rejecting the null hypothesis given that it is true (type I error). With *α*=0.05, the test is significant if |*Z*_MK_|≥*Z*_1−*α*/2_=1.96, in which case we can reject the null hypothesis *H*_0_ and conclude that there is a monotonic trend over time. Otherwise, we accept the null hypothesis and conclude that no monotonic trend exists in the data. As an example, we use this methodology to test the weights obtained using SVD and ICA in §4b, each representing the trend of one component over the 100 measurements. The MK test statistics *Z*_MK_ are computed and labelled to the right of the weights in figure 6*c*,*f*. The *Z*_MK_ of the sixth component of ICA and the fourth component of SVD are, respectively, 11.81 and 12.55, corresponding to *p*-values close to zero. This suggests that we should accept the alternative hypothesis and conclude that a monotonic trend exists in the data. Note that after decomposition, the monotonic trend is represented by a single component, while the other components, including but not limited to those shown in figure 6*c*,*f*, all have *Z*_MK_ that are smaller than *Z*_1−*α*/2_=1.96 with a significance level of 0.05, marking the absence of a monotonic trend. This example shows that SVD and ICA clearly separate the damage-related component from those corresponding to other variations, and that we can identify the damage-related component by using the MK test.

## 5. Results

We apply the three damage detection schemes described in §4 to the synthetic datasets generated with different test parameters listed in table 1, and then process the results using the evaluation framework illustrated in §3. We first compare the performance of the three methods on one synthetic scenario in §5a, and introduce the area under curve (AUC) to compare the ROC curves across different test scenarios in §2b, and comprehensively compare the three methods in §5c over test scenarios listed in table 1. The more difficult case where damage is added near a structural feature is discussed in §5d.

### (a) Evaluation of different methods in a single scenario

**Figure 7.**
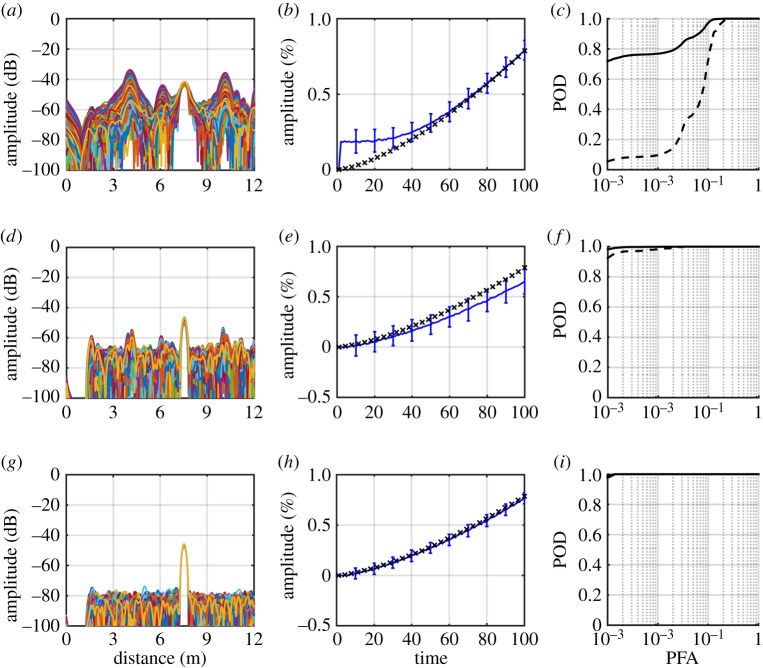
(*a*–*c*) Damage detection results using residual, (*d*–*f*) SVD and (*g*–*i*) ICA on 500 synthetic datasets containing 100 measurements collected over a 10°C temperature range, while damage grew linearly from 0 to 1% CSAL at 7.5 m. (*a*) Five hundred baseline subtracted residuals, each from one synthetic dataset. (*d*,*g*) Five hundred damage-related components from SVD and ICA, each from the decomposition of one synthetic dataset. (*b*) Amplitudes of baseline-subtracted residual at damage location over time (blue) and true damage amplitude (black). (*e*,*h*) Weights from SVD and ICA (blue), along with true damage amplitude (black). (*c*,*f*,*i*) ROC curves (solid) and the one-sided 95% lower one-sided prediction bound (dashed), that estimated the POD in 95% of the cases. (Online version in colour.)

We compare the damage detection results of residual processing, SVD and ICA side-by-side on the same test scenario previously discussed in §§3d and 4b. Here, instead of processing a single dataset generated from that test scenario, we process 500 synthetic datasets with the same test parameters, where each dataset contains 100 undamaged measurements randomly selected over a temperature range of 10°C, superposed with damage growing linearly from 0 to 1% CSAL. Figure 7 shows the extracted damage signatures, their amplitudes over time, and the ROC curves.

Figure 7*a* shows the baseline subtracted residuals, obtained using the procedure described in §4a on the 500 datasets. The same datasets are also processed with SVD and ICA with the decomposition in §4b and then the MK trend test in §4c, and the resulting damage-related components are shown in figure 7*d*,*g*.

Figure 7*a*,*d*,*g* all show a clear peak at 7.5 m, where the damage is introduced. However, the damage signature in the residual signal in figure 7*a* is sometimes masked by the large residual noise, especially at structural features at 4 m and 10 m. The components obtained from SVD figure 7*d* and ICA figure 7*g* both clearly show damage features that are much larger than the ambient noise; the SNR in the ICA damage component (figure 7*g*) is on average 30 dB higher than that of the residual (figure 7*a*), and is roughly 10 dB higher than that of SVD (figure 7*d*), indicating that the performance is best with the ICA method.

Figure 7*b* shows the residual amplitude over time at the damage location. For the baseline subtraction method, the damage amplitude is usually represented by the maximum or the root mean square (RMS) of the residual signal; provided the baseline is perfectly removed, those measures will accurately track the damage growth. However, in the presence of EOC variations, these approaches are prone to errors resulting from imperfect baseline removal. Therefore, we process the residual amplitudes at each location separately using the MK trend test, then plot the residual amplitudes at the location associated with the highest *Z*_MK_ as the representation of damage growth. The error bars show the standard deviations of the amplitudes. The true damage amplitude is also shown in figure 7*b* as black crosses for comparison. The residual amplitude is initially masked by the noise floor but approximates the true damage amplitude (black crosses) as damage grows and produces a reflection larger than the noise. Note the error bars in figure 7*b*,*e*,*h* are the results of processing the whole 100 measurements, not of processing the observations up to that particular time step. Therefore, it shows how the history of damage growth would be seen after the 100 measurements over the growth to 1% CSAL. The detectability of different levels of damage and the effect of changing the number of readings are considered in §5c. Figure 7*e*,*h* shows the weights obtained from SVD and ICA, respectively, both of which show clear monotonic increasing trends with time. The weights of SVD in figure 7*e* follow a similar growth pattern as the true damage progression, but the growth rate is underestimated by about 15%. The weights of ICA in figure 7*h* closely follow the true damage amplitudes, and show the smallest variation of the three, providing better tracking of the damage growth.

We then obtain the ROC curves by processing the extracted damage signatures with the procedure described in §3d. Figure 7*c*,*f*,*i* shows the ROC curves for, respectively, residual processing, SVD and ICA. The ROC curves of the 500 synthetic datasets are ‘vertically‘ averaged (giving the averaged POD at a given PFA) [18] and plotted as a solid line, while the dashed line shows the lower one-sided 95% prediction bound of POD, indicating that with a 95% probability future observations will fall above this value. The ROC curves demonstrate the performance in the case of linear damage growth (giving nonlinear growth in reflection amplitude) to a maximum 1% CSAL with 100 readings over a 10°C temperature range. The baseline-subtracted residual successfully indicates the presence of damage most of the time, with some false alarms caused by the residual noise that often masks the damage signature. The wide gap between the average performance and the lower 95% prediction bound suggests that it is also less robust to the temperature variations. As an example, along the dashed curve in figure 7*c* that represents the 95% one-sided lower prediction bound, if a POD of 90% is required, then the corresponding PFA is 13%. By contrast, with SVD and ICA a lower 95% prediction bound POD of 99% is obtained at PFA of 1.8% and 0.12%, respectively.

### (b) Comparison of ROC curves across different scenarios

Section 5a demonstrates the operation of our proposed evaluation framework when comparing the three different damage detection methods in a single damage growth—EOC variation case (1% linear CSAL growth and 10°C temperature range). However, ROC curves are a two-dimensional depiction of classifier performance, and are difficult to compare across multiple scenarios. Therefore, we use the area under the ROC curve (AUC) [39] as a scalar metric to facilitate the comparison of the performance of the different methods across multiple scenarios. AUC is a single scalar metric whose value lies between 0 and 1, representing the proportion of the area of the unit square. An AUC of unity corresponds to a perfect detector and 0.5 corresponds to a random guess.

**Figure 8.**
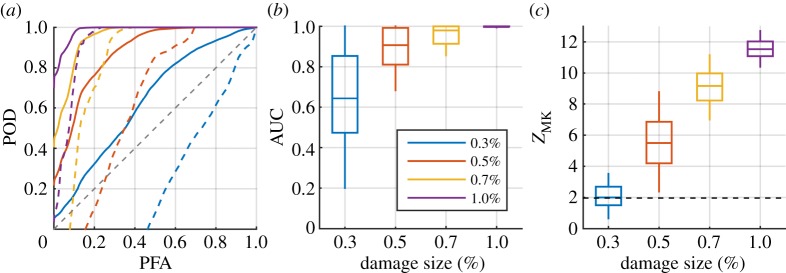
Comparison of damage detection using baseline subtracted residual across different scenarios; different colours represent synthetic datasets generated with different damage growth rates over 100 records over a 10°C temperature range. The purple curves are results of the same case shown in figure 7*c*. (*a*) ROC curves (solid lines) and lower one-sided 95% prediction bound (dashed lines), (*b*) box plots of area under curve with boxes indicating interquartile and whiskers indicating 90% two-sided prediction interval and (*c*) box plots of *Z*_MK_ of the weights. (Online version in colour.)

Here, we demonstrate AUC on the results of residual processing on four synthetic datasets generated with different damage severities, including the one processed in §5a. For each of the four scenarios, 500 synthetic datasets were generated, each containing 100 randomly selected experimental signals collected over a 10°C temperature range. Figure 8 shows the four cases with different colours where damage grows linearly from zero to, 0.3%, 0.5%, 0.7% and 1.0%.

Figure 8*a* shows the mean ROC curves on a linear PFA scale as solid lines and their 95% lower one-sided prediction bounds as dashed lines. Unsurprisingly, the larger the damage growth rate is, the more the ROC curve approaches the top left hand corner of the plot, and the smaller is the variance. Figure 8*b* shows four box plots of AUCs corresponding to the ROC curves in figure 8*a*, where the box, the horizontal line, and the vertical whisker indicate, respectively, the interquartile, the median, and the 90% two-sided prediction interval of the 500 AUCs. Two-sided prediction interval is used here such that the lower bound, which is of most interest in the evaluation of the performance, is consistent with the one-sided 95% bound shown in figure 8*a*. The AUC box plots clearly show the performance improves as damage severity increases, with the boxes moving up the AUC axis, corresponding to higher POD and lower PFA, and their smaller extent along the AUC axis corresponding to smaller variance. Note that in the case of 0.3% damage growth (blue), some of the AUCs drop below 0.5, corresponding to ROC curves below the diagonal line, indicting that the performance is worse than a random guess. This happens when the extracted damage feature is similar in amplitude to the ambient noise and is much smaller than the residual noise at structural features.

Figure 8*c* shows the MK test statistic *Z*_MK_ indicating the presence of a monotonic trend. The indications from *Z*_MK_ are consistent with those from the ROC curves and AUCs. As the damage growth over the 100 readings increases, the *Z*_MK_ test statistic increases, and at damage of 0.5% or higher CSAL after 100 readings, in over 95% of the 500 cases *Z*_MK_ exceeds the 1.96 threshold associated with a significance level of 0.05, as marked as a dashed line in figure 8*c*. As the trend of *Z*_MK_ is consistent with that of AUC, in the next section, we primarily use AUC to summarize the results and compare performance across different scenarios.

### (c) Evaluation of different methods across different scenarios

**Figure 9.**
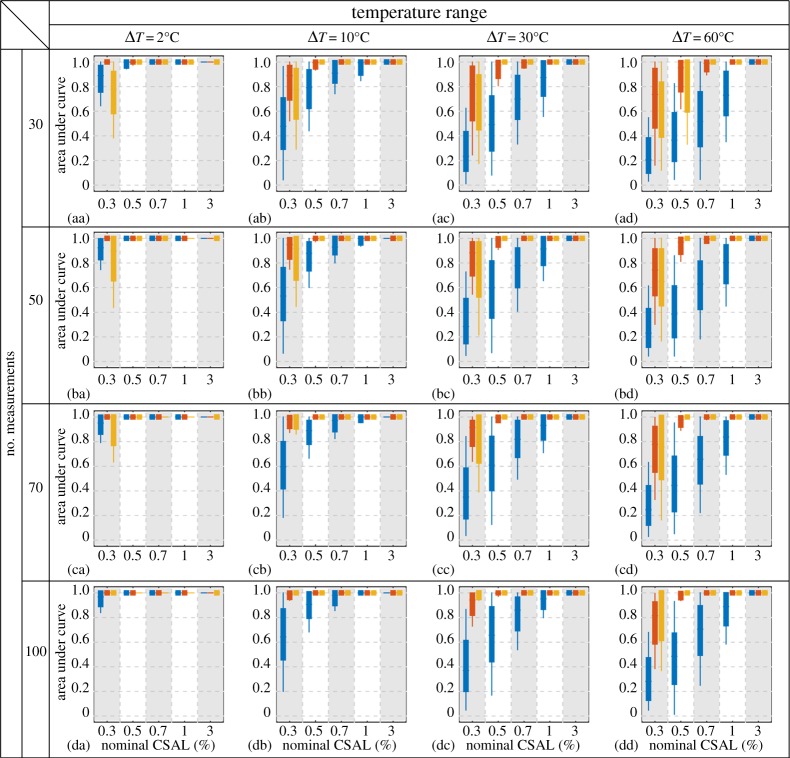
Box plots of AUCs on synthetic datasets generated with different test parameters. The boxes indicate the inter-quartiles (25% to 75%) and the whiskers extend to the 5% and 95% marks. Rows: different numbers of readings. Columns: different temperature ranges. Horizontal axis: nominal CSAL at the end of the linear damage growth. The blue, red and yellow boxes in each stripe represent results of, respectively, residual processing, SVD and ICA. (Online version in colour.)

We now extend the analysis to compare the three methods across scenarios with different combinations of test parameters from table 1. Figure 9 shows AUC box plots for each test scenario, where the three colours correspond to the results of residual processing (blue), SVD (red) and ICA (yellow). The four rows correspond to different numbers of readings taken during the damage growth, and the four columns correspond to different temperature ranges. Within each plot, the horizontal axis indicates the nominal CSAL at the end of the linear damage growth, where the stripes mark the results of the three methods on the same test scenario.

Unsurprisingly, with all other parameters equal, the methods achieve better performance (higher AUCs) when damage is larger, or when temperature variation is smaller. The performance also improves with increasing number of measurements taken during the damage growth. Comparing the three methods, SVD and ICA produce much better results than the conventional residual processing in all test scenarios, especially when the temperature variation is large. ICA generally outperforms SVD except in cases where damage is small (0.3% CSAL).

We can use figure 9 to assess the damage detection performance as a function of any of the three varying parameters, while keeping the other two unchanged. For example, figure 9(cc) shows the performance of detecting different levels of damage growth across 70 measurements under 30°C temperature variations. Assuming we are satisfied with a detector if its AUC exceeds 0.95, which roughly corresponds to 90% POD with 2% PFA (this level of PFA may not be practically acceptable, but is used here only to compare the methods), then at a 95% prediction level we can use SVD and ICA to detect the damage before it grows to 0.5% CSAL. On the other hand, although the residual performance improves as damage severity increases, it does not perform sufficiently well until damage grows to 3%. Similarly, comparing figure 9(ad, bd, cd, dd), we see that detecting 0.5% CSAL growth with a 95% prediction bound AUC of 0.9 requires at least 70 measurements for SVD, or at least 50 for ICA.

**Figure 10.**
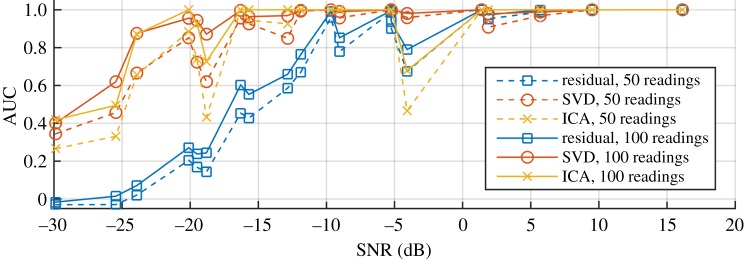
The lower 95% prediction bound of AUC of three methods against the ratio of damage amplitude over temperature noise. Dashed lines and solid lines are generated with synthetic datasets containing, respectively, 50 readings and 100 readings. (Online version in colour.)

To successfully implement these methods, it is critically important and of most interest to estimate the lower prediction limit of the damage detection performance in the practical settings of the SHM system, which bounds the worst-case performance of a future test. The results of figure 9 show that the SHM system performance improves with damage size and degrades with degree of temperature variation. It is therefore interesting to plot performance versus the SNR, which is defined as the reflection coefficient of the damage at the end of the measurement period divided by the maximum amplitude of the coherent noise resulting from the temperature range. The SNR indicates how difficult it is to detect damage in this scenario—a small SNR corresponds to the growth of small damage over a large temperature range, and a large ratio corresponds to the growth of large damage over a small temperature range. Figure 10 plots the 95% one-sided lower prediction bound of AUC against the SNR. The three colours correspond to the three damage detection methods, and solid and dashed lines represent the results from synthetic datasets of, respectively, 50 and 100 measurements. Note that different combinations of damage amplitude and temperature range may result in similar SNR. For example, three test cases with SNR of around −20 dB correspond to, respectively, 0.3% CSAL over 10°C (SNR=−18.8 dB), 0.7% CSAL over 30°C (SNR=−19.5 dB) and 1.0% CSAL over 60°C (SNR=−20.1 dB).

We can separate damage detection applications into three regimes by the SNR: with SNR larger than 0 dB, we generally obtain good detection results with any of the methods; with SNR in between 0 and −18 dB, we gain from sophisticated processing, such as SVD and ICA; with SNR smaller than −18 dB, we cannot obtain satisfactory results with any of the methods.

The anomalies in figure 10 at −4.1 dB and −18.8 dB correspond to a damage level of 0.3% over different temperature ranges, which suggests that there is a small source of coherent noise that is not suppressed by temperature compensation and this affects the detectability of small defects. This requires further investigation, but it should be stressed that 0.3% cross-section loss is more than an order of magnitude smaller than the wall loss typically detected in guided wave inspection [5]. Comparing SVD (red circles) and ICA (yellow crosses), SVD seems to be more robust to this effect.

The dashed lines and the solid lines represent results with, respectively, 50 and 100 readings over the damage growth. For a year of monitoring, 50 readings roughly corresponds to observations made weekly, while 100 readings corresponds to 2 measurements per week. Comparing the two sets of results, we can conclude that more robust damage detection can be achieved with more measurements.

### (d) Evaluation of different methods with the damage near to structural features

In previous sections, we evaluated and compared damage detection performance of the three methods on different scenarios where damage was added at 7.5 m, in a plain section of the pipe. Here, we further process synthetic datasets with damage added at other locations including ones close to structural features.

**Figure 11.**
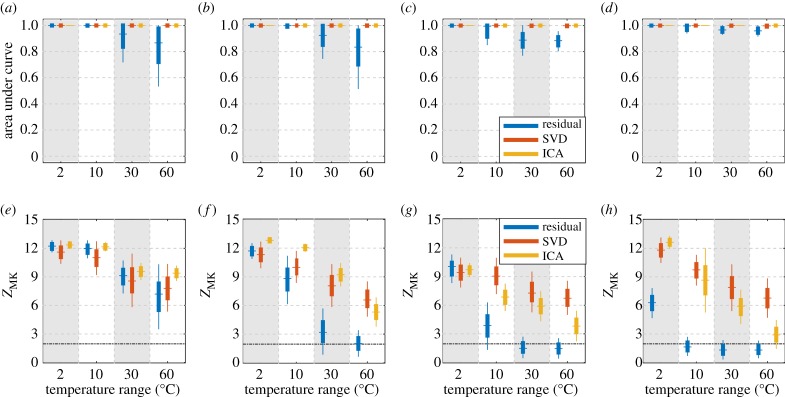
Box plots of AUC and *Z*_MK_ of detecting 1% damage growth over 100 measurements over different temperature ranges, where damage is added at four different locations in table 1. Plots (*a*,*e*) and (*b*,*f*) show results when damage is added at, respectively, 2.3 m and 11.3 m, away from structural features. Plots (*c*,*g*) and (*d*,*h*) show results when damage is added at, respectively, 6.2 m or 10.1 m, close to structural features. The dot-dash line in (*e*–*h*) shows the threshold of 1.96. (Online version in colour.)

Figure 11 shows four pairs of AUC (figure 11*a*–*d*) and *Z*_MK_ (figure 11*e*–*h*) of the three methods detecting 1% damage growth over 100 measurements. The four stripes in each plot mark temperature ranges of 2, 10, 30 and 60°C, respectively. Figure 11*a*,*e*,*b*,*f* shows results when damage is added at, respectively, 2.3 m and 11.3 m, both in plain sections of the pipe. As with the results shown in figure 9(da–dd) where damage was added at 7.5 m, here the SVD and the ICA both yield AUCs close to unity, indicating successful detection of damage with few false alarms. The residual processing also achieves AUCs close to unity when the temperature range is small (2 or 10°C), but its performance quickly degrades with increasing temperature range. The corresponding *Z*_MK_ displays the same trend as the AUC, as in figure 8.

Figure 11*c*,*g*,*d*,*h* shows the cases where damage is added at locations close to structural features. In these cases, the AUC in itself cannot represent the detection performance, and needs to be interpreted together with the *Z*_MK_. The two cases display similar behaviour, so we only discuss figure 11*d*,*h* for conciseness. Figure 11*d*,*h* corresponds to the cases where damage is added at 10.1 m, slightly after the second end reflection. Recall that we are using reverberating signals to represent a longer pipe in which the end reflections would be large features. In all four cases over different temperature ranges in figure 11*d*, the AUCs of SVD and ICA are close to unity, and the AUCs of residual processing are also over 0.9. The AUC with the residual method appears high because features tend to generate relatively large residuals; at a feature the residual is the difference between two large reflections in the baseline and current, temperature compensated signal, whereas away from features the signals are smaller so it is easier to obtain a small residual. By contrast, the *Z*_MK_ in figure 11*h* suggests that when the temperature range exceeds 10°C, the residual processing fails the MK trend test. In this case, we should conclude that no damage is detected along the pipe at any location, and should not calculate the ROC. We should also extend this interpretation to SVD and ICA, where we use the MK test to automatically determine the damage-related component: when the *Z*_MK_ of all the components are below the threshold, it should be concluded that no single component represents the damage, and that damage is not present in the dataset being processed.

**Figure 12.**
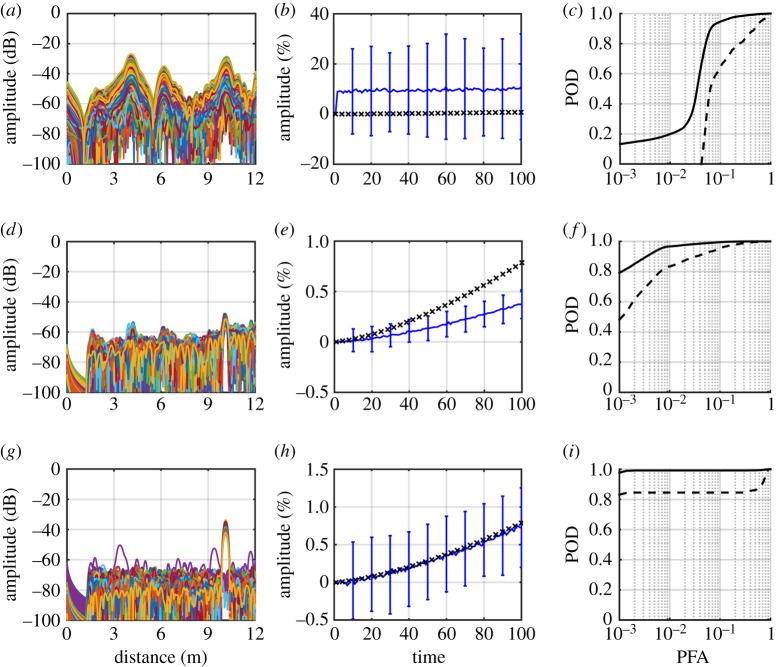
(*a*–*c*) Damage detection results using residual, (*d*–*f*) SVD and (*g*–*i*) ICA on synthetic datasets each containing 100 measurements collected over a 30°C temperature range, while damage grew linearly from 0 to 1% CSAL at 10.1 m (slightly after second end reflection). (*a*) Baseline subtracted residuals. (*d*,*g*) Damage-related components from SVD and ICA. (*b*) Amplitudes of baseline-subtracted residual at damage location over time (blue) and true damage amplitude (black). (*e*,*h*) Weights from SVD and ICA (blue), along with true damage amplitude (black). (*c*,*f*,*i*) ROC curves calculated from the damage signatures in (*a*,*d*,*g*) and the 95% lower one-sided prediction bound. (Online version in colour.)

As an example, figure 12 plots results of the three methods on synthetic datasets corresponding to the third stripes in figure 11*d*,*h*. Each synthetic dataset contains 100 measurements collected over a 30°C range, while damage grew linearly from 0 to 1% CSAL at 10.1 m (slightly after second end reflection). The residuals in figure 12*a* are dominated by the large residual errors at structural features that mask the damage signature. The weights shown in figure 12*b* display a 15% s.d., and no monotonic trend can be identified by the MK test. Figure 12*c* shows the 95% lower prediction bound of ROC curve with large PFAs even for small PODs.

Figure 12*d*,*e*,*f* shows the results of SVD, which successfully extracts the damage signature and suppresses the benign features. Although the components shown in figure 12*d* are still noisy, we can nevertheless easily identify the damage signature at 10.1 m, which is at least 6 dB larger than the noise elsewhere. Correspondingly, the ROC curve in figure 12*f* shows that a 95% lower prediction bound POD of 90% corresponds to a PFA of 3.3%. The weights in figure 12*e* significantly underestimate the damage growth rate, but its trend is qualitatively consistent with the true damage growth.

The ICA components in figure 12*g* show very clear damage signatures in most cases. However, on four of the 500 datasets (e.g. the purple line in figure 12*g* with peaks at 3.5 m and 9.5 m), ICA fails to extract the damage component and yields components corresponding to noise from other random variations. These outlier cases produce the large variations in the weights as shown in figure 12*h*, and the large drop in the 95% prediction bound of POD in figure 12*i* from its mean. When the temperature range increases from 30°C to 60°C, the proportion of the outlier cases increases from 1% to 20%, producing a significant degradation of *Z*_MK_ as can be seen in figure 11*h*. As a consequence, we obtain better results using SVD than using ICA when damage was added close to a structural feature under large temperature variations. A probable cause of the outliers is that ICA is essentially an optimization process to find a set of components that minimize the mutual information, and is therefore prone to local minima. As the temperature range increases, the damage signature becomes a smaller portion of all the variations in the data, so the optimization is more likely to get stuck in local minima.

## 6. Experimental validation

We now validate the evaluation framework on experimental data monitoring actual damage growth. A flat-bottom hole was drilled on the same pipe specimen shown in figure 1*a* at 2.3 m to the right of the PIMS transducer ring. The hole was 4 mm deep and was extended from 1 mm to 7 mm diameter in 1 mm steps, simulating progressively growing damage from 0 to roughly 0.5% CSAL. At each damage step, the pipe was heated to 90°C and naturally cooled to 30°C, and data were collected over the temperature cycle as described in §2. Data from the last temperature cycle prior to introducing the hole, together with that from the cycles after each damage set was used, giving a total of 338 measurements over the damage growth. This gave a reasonable approximation to linear damage growth from 0 to 0.5% CSAL.

As with the synthetic datasets, we assess the performance of damage detection over different monitoring records under different levels of temperature variation. For temperature ranges from 10 to 60°C in 10°C steps, we randomly selected *n* signals from each of the eight cycles, forming datasets of 8*n* monitoring records over the 0.5% damage growth, where *n*=1, 4, 6 and 9 correspond to 8, 32, 48 and 72 records, respectively. Note that here we retain the chronological order of the selected measurements from different damage steps, but the measurements from the same damage step are randomly ordered, such that the temperature associated with the measurements varies randomly. This random selection process was repeated 50 times for each combination of test parameters (temperature range and number of records) to generate statistics.

**Figure 13.**
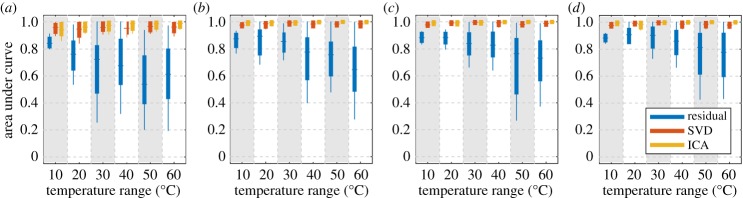
Results of baseline-subtracted residual (blue), SVD (red) and ICA (yellow) on experimental records. Plots (*a*–*d*) Correspond to the results of using, respectively, 8, 32, 48 and 72 measurements. The horizontal axis indicates the temperature range over which measurements are selected. (Online version in colour.)

Figure 13 shows the results of residual processing (blue), SVD (red) and ICA (yellow) on different numbers of experimental records over different temperature ranges. Figure 13*a*–*d* corresponds to the results using, respectively, 8, 32, 48 and 72 measurements, while the horizontal axis in each plot indicates the temperature range over which those measurements are selected.

The baseline-subtracted residual performs poorly when detecting the 0.5% damage, with AUCs mostly lower than 0.9, and shows only small improvement when more experimental records are processed. In comparison, both SVD and ICA perform reasonably well using 32 records, and better with more records; the performance generally degrades as the temperature range increases. Overall, ICA outperforms SVD, especially when many monitoring records are available.

Comparing figure 13 with the results using synthetic data in figure 9, the performance is qualitatively consistent. As with the synthetic data, the component methods perform significantly better than the residual method, with ICA generally the better of the two, indicating that the proposed framework of using experimental data on an undamaged system coupled with predicted damage signals is effective. The performance on experimental data is slightly better than previous results with synthetic datasets as shown in §5c, especially at large temperature ranges. This is probably partly because we only processed the data up to and including the first end reflection, as would be done in practice; this reduces the number of large, benign reflectors in the signal, and hence the likelihood of false calls.

## 7. Conclusion

We have proposed an evaluation framework to assess the damage detection performance of guided wave SHM systems under varying EOCs. By synthetically superposing damage reflection signals from a growing defect onto experimental signals from an undamaged structure measured under practical environmental variations, we can evaluate the performance of an SHM system under various scenarios of interest. This enables the effect of temperature range over which measurements are taken, damage location, damage amplitude, the number of observations over the monitoring period, etc., to be studied. Moreover, by generating multiple datasets with randomly selected experimental records, we can statistically evaluate the performance in these practical settings. The superposition methodology has been validated in test cases with different degrees of complexity and has been shown to be sufficiently accurate. We have demonstrated the framework using undamaged data taken on an 8 inch schedule 40 pipe in the laboratory under up to 60°C temperature variations.

The damage detection performance was assessed by generating the ROC curve, which plots the POD against the probability of false alarm (PFA). Three damage detection methods, conventional baseline-subtracted residual processing, SVD and ICA, were evaluated. Under all investigated circumstances, the component methods performed significantly better than the residual method, with ICA generally better than SVD except for a small number of outliers. For all three methods, the performance improves with larger damage size and more observations, and degrades with larger range of temperature variations. We then validated the results using experimental data monitoring a flat-bottom hole with growing diameter, the results being consistent with the synthetic data.

The results show that we can use undamaged data from any installed monitoring system to predict its damage detection performance. This makes it possible, for example, to assess whether a defect of a given size at a particular location would be detected reliably at a given data collection frequency. This enables the system to be tuned to meet particular requirements and the performance demonstration will be valuable in building safety cases.

## Data accessibility

The experimental signals described in §2 can be accessed at https://doi.org/10.5281/zenodo.250179. These data are made openly available with citation under a Creative Commons CC-BY license.
